# Evaluation of the Shelf life of Ready-to-Eat Fresh Bamboo Sprouts (*Phyllostachys edulis*) Packaged in a Modified Atmosphere or Vacuum: A Comparative Study

**DOI:** 10.3390/antiox13020185

**Published:** 2024-02-01

**Authors:** Vincenzo Sicari, Rosa Tundis, Rosa Romeo, Antonella Reitano, Emilia Lucia Belsito, Antonella Leggio, Monica Rosa Loizzo

**Affiliations:** 1 Department of Agraria, “Mediterranea” University of Reggio Calabria, Cittadella Universitaria, Località Feo di Vito, 89124 Reggio Calabria, RC, Italy; vincenzo.sicari@unirc.it (V.S.); rosa.romeo@unirc.it (R.R.); 2 Department of Pharmacy, Health and Nutritional Sciences, University of Calabria, 87036 Rende, CS, Italy; rosa.tundis@unical.it (R.T.); emilialucia.belsito@unical.it (E.L.B.); antonella.leggio@unical.it (A.L.); 3 Department of Business and Legal Sciences, University of Calabria, 87036 Rende, CS, Italy; antonella.reitano@unical.it

**Keywords:** *Phyllostachys edulis*, modified atmosphere packaging, vacuum, shelf-life, bioactives evolution, antixidant

## Abstract

During the last decades, the consumption of bamboo sprouts (*Phyllostacys edulis*) has increased because they are considered a “superfood”. However, this product is characterized by a short shelf life due to the deterioration in quality parameters. The aim of this study was to investigate the application of two modified atmosphere packaging (MAP) systems (MAP1: 2% O_2_, 5% CO_2_, 93% N_2_ and MAP2: 3% O_2_, 7% CO_2_, 90% N_2_) to fresh-shelled ready-to-eat bamboo sprouts and compare these packaging systems with vacuum packaging during storage for 28 days at 4 °C using heat-sealable polyamide and polyethylene (PA/PE) trays. Several chemical-physical parameters (moisture content, water activity, pH, headspace composition, and firmness) were monitored, as well as CIELab colorimetric parameters and microbial growth. The quantification of selected organic acids was performed via UHPLC. Mathematical kinetic models were applied to study the evolution of total phenol (TPC), flavonoid (TFC), and carotenoid content (TCC) during storage. The evolution of antioxidant potential investigated by ABTS, DPPH, and β-carotene bleaching tests was also assessed. Results showed that at the end of the storage period, significant variations in the colorimetric parameters are detectable between the sprouts apical portion and the basal one, regardless of both applied MAPs. A linear reduction in both DPPH and ABTS radical scavenging activity was evidenced during storage, regardless of the type of packaging applied. In DPPH test samples packaged in MAP after 28 days of storage, they retain good antioxidant activity, whereas in vacuum, this activity is reduced by 50% compared to the initial value (IC_50_ values from 24.77 to 32.74 μg/mL and from 24.77 to 71.12 μg/mL for MAP2 and vacuum, respectively).

## 1. Introduction

Bamboo is a plant widely cultivated in China, India, and the Philippines [1]. The sprouts, which are considered a delicacy, are consumed fresh, fermented, canned, or roasted, or in traditional recipes such as “kupe”, “Usoi ooti”, and “lumpa” [1,2,3]. Bamboo sprouts, which are the tender stems emerging from the nodes of the (pseudo-)rhizome of bamboo plants, are wrapped in protective, inedible leaf sheaths, with the edible portion consisting of meristematic cellular tissue with regions of rapid division and cellular differentiation [2,4,5].

Bamboo is known for its health properties, including antioxidant properties, cholesterol-lowering, anticancer, cardiovascular, and neuroprotective effects [4,6]. These activities are related to the high level of functional micronutrients such as high-quality proteins, vitamins, beta-carotene, phenolics, and phytosterols [7]. Moreover, the sprouts are an excellent source of fiber while containing low concentrations of sugars and fats [8]. Indeed, a significant increase in its consumption was observed in the last decade.

During storage, as with other vegetables, bamboo sprouts can undergo alteration phenomena such as loss of texture, microbial growth, browning process, and formation of off flavors [9,10,11]. For the above-mentioned reasons, the shelf life of bamboo sprouts is limited due to the deterioration of quality-related attributes, including sensory characteristics, from the postharvest procedure. The application of an adequate packaging solution capable of counteracting the deterioration of quality and responding to market needs by extending the shelf life of fresh sprouts has gained the attention of researchers. 

Several studies investigated the process of quality deterioration in different species of bamboo sprouts [4,9,10,11,12]. Most of these studies suggest that the shelf life of fresh bamboo sprouts at room temperature in non-MAP (modified atmosphere packaging) conditions is limited to one day due to the rapid change in color and consistency. At the same time, a possible solution is the storage of fresh sprouts in MAP at a low temperature. 

The use of MAP has become increasingly common in the last ten years. This packaging system is based on the application of a gas mixture including nitrogen, carbon dioxide, and sometimes oxygen at reduced concentrations. This postharvest technique, both under active and passive conditions, in combination with cold storage, is very useful for extending the shelf life of ready-to-eat products [13,14]. In fact, products packaged by MAP maintain their taste, texture, and appearance. For these reasons, it has been successfully utilized to extend the shelf life of fresh vegetables and fruits, thus prolonging the distribution chain and diminishing the need for centralized production. Moreover, MAP is inexpensive, easy to apply, and suitable for a wide range of packaging machinery and production venues [15,16]. The definition of the appropriate atmosphere inside the packaging depends mainly on the respiration rate of the product, the temperature, the weight of the filling, the surface of the film, and its permeability [17].

However, even though a wide literature on the use of MAP for fresh-cut fruit and vegetables is available [16], scientific information on its application for bamboo sprouts remains sparse.

Vacuum packing is a method of packaging that removes air from the package prior to sealing. It reduces atmospheric oxygen, limits the bacterial fermentation process, and prevents the evaporation of volatile components. However, with vegetables and fruits, several problems with texture were observed [18,19].

Therefore, the purpose of this work was to investigate the impact of vacuum and MAP packaging systems on the physico-chemical and microbiological parameters as well as the bioactive phytochemical content and the antioxidant activities of the fresh bamboo sprouts stored at 4 °C for 28 days. 

## 2. Materials and Methods

### 2.1. Chemicals and Reagents 

All reagents were obtained from Sigma Aldrich (Milan, Italy). Solvents of analytical grade were purchased from VWR International s.r.l. (Milan, Italy). Oxalic, tartaric, malic, citric, succinic, and fumaric acids were purchased from Fluka (Steinheim, Germany). 

### 2.2. Plant Materials 

Fresh bamboo sprouts (*Phyllostacys edulis* J. Houz) were obtained from Falco farm (Corigliano Calabro, Cosenza, Italy). Sprouts, devoid of any form of known injury, were collected randomly on a total area of approximately 20 hectares in the morning and transported at 25 ± 3 °C to the Department of Pharmacy, Health and Nutritional Science, Food Science Technology Laboratory (University of Calabria, Italy) within 3 h. 

Sprouts were checked for insect contamination, manually peeled to remove the sheath, and washed with a chlorinated solution (300 mg/L). Then, samples were selected based on criteria as follows: 0.30–0.70 kg of weight, 15–25 cm of length, and 3–5 cm basal of diameter. Before analysis, 2 cm of basal sprout was cut. 

### 2.3. Sample Preparation and Experimental Design

To evaluate the impact of the packaging system on the shelf life of fresh peeled bamboo sprout, samples were randomly divided into three groups and then stored in:

- polypropylene (PP) heat-sealed trays sealed with a polyamide/polyethylene (PA/PE) film. Inside MAP1, there was the following mixture of gases: 2% O_2_, 5% CO_2_, 93% N_2_.
- polypropylene (PP) heat-sealed trays sealed with a polyamide/polyethylene (PA/PE) film. The following gas mixture was inserted in this group 3% O_2_, 7% CO_2_, 90% N_2_ (MAP2).
- vacuum packed in food-grade multi-layer polythene (PE) bags measuring 350 × 180 × 150 mm (OLPACK 510, Interprise Breussel S.A., Belgium).

Packaged samples were stored at 4 ± 1 °C for 28 days. Each treatment was conducted in triplicate and samples were analyzed the day of the treatment (day 0) and thereafter every 7 days for a total of 28 days of storage (7, 14, 21, and 28 days).

Unpacked bamboo sprouts have a very short shelf life (2–3 days). For this reason, the results of the tests conducted on these sprouts are not reported in the work.

### 2.4. Physical Quality Parameter Analysis

For each packaging system and for each sample collection, weight loss was measured. The weight of each sprout was measured with a precision balance (model: Sartorius™ BCE2202-1S, Thermo Fisher Scientific Inc., Monza, Italy) with a least count of 0.001 g.

The loss in weight was expressed as a percentage of the original fresh weight. For moisture determination, the procedure 934.06 AOAC was adopted [20].

Samples (5 g) were weighed into the moisture dishes, oven dried at 105 °C for 3 h to a constant weight and transferred into a desiccator to cool. After that, samples were weighed using a precision balance (model: Sartorius™ BCE2202-1S, Thermo Fisher Scientific Inc., Monza, Italy). Moisture was determined in triplicate for each sample. Moisture content was calculated through differences in weight before and after moisture drying to a constant weight, and the value was expressed as a percentage moisture content.

For other variables, sprout aqueous extracts were prepared by adding 10 mL of distilled water to 1 g of sprout, and then samples were homogenized using an Ultra-Turrax T-25 (Janke and Kunkel, IKA-Labortechnik, Staufen im Breisgau, Germany).

The water activity (a_w_) of sprouts was measured by an Aqualab LITE v6 hygrometer (Decagon devices Inc., Washington, DC, USA). The pH values were measured at room temperature (25 °C) using a pH meter (Crison Basic 20, Barcelona, Spain).

### 2.5. Evolution of Color Parameters during Storage

The color change of the basal and apical sections of the sprout was measured at 25 °C using a PCE CSM-4 colorimeter (PCE, Lucca, Italy) to obtain the color according to the CIEL a* b*method [21] and expressed as L* (lightness) and C* (chroma) values. Data were expressed as higher saturation of color, or Chroma (C*) (1).

(1) $$\mathrm{Chroma}\;(C*)=\sqrt{{a^{*}}^{2}{-b^{*}}^{2}}$$

To evaluate the overall change in color, the CIELab parameter was calculated following Equation (2) [22]:

(2) $$\text{△}E\;\sqrt{{{(L}_{0}^{*}-L^{*})}^{2}+{{(a}_{0}^{*}-a^{*})}^{2}{{(b}_{0}^{*}-b^{*})}^{2}}$$

Moreover, the Browning index (BI) was calculated following the procedure previously described [21].

### 2.6. Firmness Analysis

The firmness of sprouts apical and basal sections (2 cm) were measured using a texture analyzer (TA-XT Plus, Stable Micro Systems Ltd., UK) equipped with a standard cylindrical probe of 2 mm in diameter, following the parameters: load cell = 1 kg, pre-test speed = 2.0 mm/s, test speed = 1.0 mm/s, post-test speed = 2.0 mm/s, compression degree = 70%, time = 5.0 s, and trigger force = 0.05 N. Each sample was measured 10 times, and the results regarding the peak force of compression (N) were elaborated using Exponent software 6.1.4.0 (Stable Micro Systems Ltd., Godalming Surrey, UK) for data acquisition and integration [23].

### 2.7. Headspace Composition

Before the opening, the headspace composition inside envelopes and packages was determined using a CheckPoint handheld Gas Analyzer (PBI Dansensor, Milan, Italy) following the procedure previously described [24].

The gas analysis was conducted with a needle inserted through a septum previously fixed to the envelopes and packages. The results were given as oxygen and carbon dioxide concentrations (%).

### 2.8. Microbiological Analysis

The microbial growth in stored sprouts was analyzed following the method of Li et al. [25], with slight modifications. Sampling sprouts were performed under aseptic conditions. Bamboo sprouts (10 g) and sterile Ringer’s solution (90 mL) were homogenized in a stomacher bag filter and homogenized with a Bag Mixer 400CC (Interscience, Puycapel, France). An aliquot of each dilution was plated in triplicate on various selective agars. The total bacterial count (TBC) was determined in Plant Count Agar, Oxoid, and incubated at 25 ± 2 °C for 48 h. The analysis of yeast and molds required DRBC (Dichloran Rose Bengal Chloramphenicol) agar base plates and an incubation time of 48 h at 28 ± 2 °C. Microbiological data (TBC) and total yeast and mold count (TYMC) were expressed as the logarithms of colony-forming units per gram (log CFU/g). Each test was performed in triplicate.

### 2.9. Evaluation of Organic Acids during Storage

The content of organic acids (oxalic, tartaric, malic, citric, succinic, and fumaric acids) was determined following the method previously reported by Giuffrè et al. [26], with some modifications. The homogenized bamboo sprout was 10-fold diluted with ultrapure water, and after centrifugation at 4032× *g* for 10 min, the supernatants were separated and filtered through a 0.45-µm PTFE syringe filter Supelco (Supelpco, Milan, Italy).

The quantification of specific compounds was carried out by a Knauer chromatograph equipped with a UV detector operated at 210 nm and coupled with an Acclaim OA5 column (4 mm i.d. × 250 mm length × 5 µm particle size). A mobile phase of 0.2 M KH_2_PO_4_ was used for the separation with a flow rate of 0.6 mL/min. External standard calibration curves was used for the identification and quantification of components. Five injections were made for each calibration level. For the linear regression of the curves of external calibration standards, *r*^2^ values were between 0.997 and 0.999. Data processing was carried out with the support of Clarity Chromatography Software 8.1 (itdesign GmbH, Tübingen, Germany), and results were expressed as mg of acid/100 g of sample.

### 2.10. Extraction Procedure

Sprouts packaged in different packaging systems (250 g) were subjected to an ultra-sound-assisted maceration process using ethanol/water (8:2 *v*/*v*, 700 mL) as solvent in a Branson model 3800-CPXH water bath (Branson, Milan, Italy) with a frequency of 40 kHz at 25 °C for 35 min. The extraction procedure was repeated five times and after each extraction cycle, solutions were filtered and the solvent was evaporated.

The obtained extracts were used for phytochemical content, Ultra-High-Performance Liquid Chromatography (UHPLC) analysis of fresh sprouts, and antioxidant activity evaluation.

### 2.11. Total Phenol, Flavonoid, and Carotenoid Contents and UHPLC Analysis of Fresh Sprout

Extracts obtained by a previously reported procedure (paragraph 20.10) were subjected to phytochemical analysis.

The total phenol content (TPC) of bamboo sprouts was monitored during shelf life using the spectrophotometric method proposed by Folin-Ciocalteu, as previously reported [27]. Extract (1.5 mg/mL, 100 μL) was mixed with Folin-Ciocalteu reagent (500 μL) and distilled water (1000 mL). After 10 min of incubation, 1.5 mL of 20% sodium carbonate was added, and the mixture was incubated at 25 °C for 60 min. The absorbance was measured at 765 nm using a UV-Vis (Jeneway 6003, Carlo Erba, Milan, Italy). TPC was expressed as mg of chlorogenic acid equivalents (CAE)/g of fresh sample.

The total flavonoid content (TFC) was determined using spectrophotometric quantification of the flavonoid-aluminum complex [27]. Sprout extract (1.5 mg/mL) was mixed with aluminum chloride solution (2%) in a 1:1 ratio and incubated at 25 °C for 15 min. The absorbance was measured at 510 nm, and TFC was expressed as mg quercetin equivalents (QE)/g of fresh sample.

The total carotenoid content (TCC) was determined following the procedure described by Sicari et al. [21]. Sprout extract was mixed with a NaCl 5% solution. The mixture was centrifuged (at 2268× *g* for 10 min). The supernatant (100 µL) was diluted with 900 mL of n-hexane, and absorbance was measured at 460 nm. TCC was expressed as mg β-carotene/g of fresh sample.

Ultra-High-Performance Liquid Chromatography (UHPLC) PLATINblue (Knauer, Berlin, Germany) with a PDA-1 (photo diode array detector) was used for quantification of mainly phenolic acids and flavoinoids occurring in fresh bamboo sprouts at day 0. The analytical conditions have been previously reported by Tundis et al. [27].

#### Bamboo Sprouts Bioactive Compounds Evolution during Storage

The mathematic kinetic model is a very useful technique for investigating the impact of processing on food quality. The first-order degradation kinetic was applied following the equations:

−ln (C_t_/C_0_) = kt (3)

t^1/2^ = ln(2)/k (4)

where C_0_ is the TPC, TFC, or TCC at day 0, and C_t_ is the concentration of phytochemicals at reaction times (7, 14, 21, 28 days of storage) [28].

### 2.12. Bamboo Sprouts Antioxidant Activity

Several methods have been described to investigate antioxidant activity. Most of them are based on the measurement of the capacity of antioxidant compounds to act as radical scavengers. In this study, in vitro ABTS, DPPH, and β-carotene bleaching and FRAP tests were performed.

The radical scavenging activity was investigated by ABTS and DPPH test assays as previously described [27]. In the ABTS test, to obtain a solution of ABTS^+^ radical cation, ABTS solution (7mM, 50 mL) and potassium persulphate (2.45 mM, 500 μL) were mixed and left to react overnight. After that, the solution was diluted with ethanol to reach a final absorbance of 0.70, measured at 734 nm. For the test, extract at different concentrations from 1 to 400 μg/mL (25 μL) was mixed with 2000 mL of diluted ABTS^+^ solution and left to react for 6 min at room temperature before measuring the absorbance at 734 nm.

In the DPPH assay, extract (1000–15.75 μg/mL) was mixed with a DPPH solution (1.0 × 10^−4^ M, 1500 μL). After half an hour at room temperature, the absorbance was measured at 517 nm (UV-Vis Jenway 6003 spectrophotometer, Carlo Erba, Milan, Italy). Results were expressed as extract concentrations able to scavenge 50% of DPPH radicals (IC_50_).

The β-carotene bleaching test was used to evaluate the ability of bioactive compounds in bamboo sprouts to counteract lipid peroxidation. Briefly, 1 mL of β-carotene solution (0.2 mg/mL in chloroform) was added to 20 μL of linoleic acid and 200 μL of 100% Tween 20. After evaporation of chloroform and dilution with water, 5 mL of the emulsion were transferred into different test tubes containing 200 μL of samples in 70% ethanol at concentrations in the range of 2.5–100 μg/mL. After incubation at 45 °C for 30 min, the absorbance was read at 470 nm [21].

The FRAP test is based on the redox reaction that involves the TPTZ (2,4,6-tripyridyl-striazine)-Fe^3+^ complex. FRAP reagent was mixed with a sample dissolved in ethanol at a concentration of 2.5 mg/mL. After half an hour of incubation at 25 °C, the absorbance was read at λ = 595 nm using a UV-Vis Jenway 6003 spectrophotometer (Carlo Erba, Milan, Italy) [21].

### 2.13. Statistical Analysis

Results were expressed as means of three different experiments ± standard deviation (S.D.). All data were analyzed using one-way analysis of variance (ANOVA) with SPSS 17.0 (SPSS Inc., Chicago, IL, USA) statistical software. Significant differences were calculated according to Tukey’s multiple range tests. Differences at *p <* 0.01 (**) and *p <* 0.05 (*) were statistically significant. GraphPad Prism version 4.0 (San Diego, CA, USA) was used to calculate the concentration-response curve and the inhibitory concentration 50% (IC_50_). Principal Component Analysis (PCA) was applied using SPSS software for Windows, version 17.0 (Chicago, IL, USA).

## 3. Results and Discussion

### 3.1. Evolution of Fresh Bamboo Sprouts Quality Parameters during Storage

Up until now, the evidence for the shelf life and storage of fresh bamboo sprouts under an active MAP at low temperature has remained unclear. At the same time, Caleb et al. [29] evidenced that the optimized ratio between O_2_ and CO_2_ for a wide variety of fruits and vegetables should be: O_2_ (3–19%) and CO_2_ (1–23.28%). Hence, we hypothesized that the active MAP can be a suitable quality preservation technique to prolong the shelf life of ready-to-eat bamboo sprouts at 4 °C. Therefore, the present study investigated the extension of the shelf life of ready-to-eat bamboo sprouts using active MAP at 4 °C. Selected physico-chemical and microbiological parameters were selected as bamboo sprout quality markers and herein investigated.

The weight reduction of fresh fruit and vegetables is due to the loss of water through respiration and transpiration. Furthermore, the type of packaging increases the humidity inside the package and reduces weight loss [30]. Moradinezhad et al. [31] reported that the use of vacuum packaging was reported to significantly reduce weight loss compared to control samples; they stated that the modified atmosphere around the pomegranate fruit delayed ripening and reduced weight loss.

**Figure 1:**
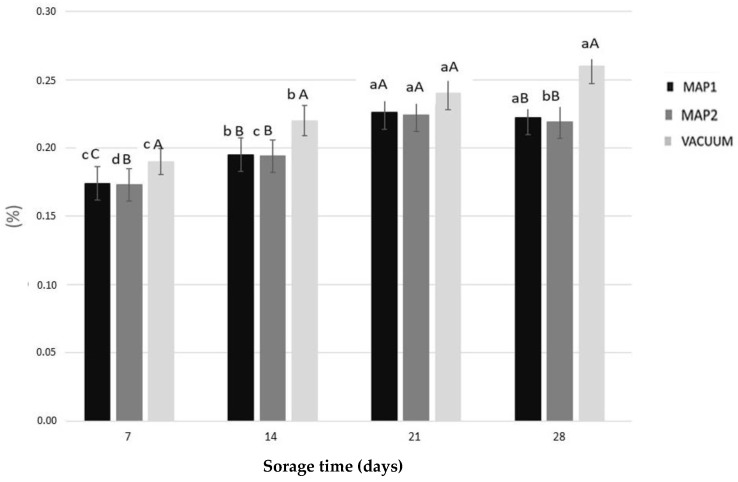
Weight loss (%) in fresh bamboo sprouts subjected to different packaging systems. MAP: modified atmosphere packaging. MAP1: 2% O_2_, 5% CO_2_, 93% N_2_; MAP2: 3% O_2_, 7% CO_2_, 90% N_2_. Data are reported as mean ± standard deviation (n = 3). Results followed by different capital letters indicatedifferences among the samples. The different lowercase letters indicatedifferences during storage. Differences within and between groups were evaluated by a one-way ANOVA followed by Tukey’s test (*p* < 0.01). Results followed by different letters are highly significantly different at *p* ≤ 0.01.

The weight loss of freshly peeled bamboo sprouts packaged using different systems is reported in Figure 1, where it is possible to see that the weight loss has a comparable trend in both modified atmosphere packaging (MAP1 and MAP2) samples. On the contrary, in the vacuum-packaged sample, a significant weight loss (approximately −0.25% at t = 28 days) was observed. Weight loss is the consequence of the peeling operations and indicates the high transpiration of the sprouts.

Kleinhenz et al. [10] reported that *B. oldhamii* sprouts, which had a weight loss of about −5% from their original weight at the beginning of the experimental process, were visually inacceptable, and this condition was critically influenced by the storage temperature and packaging materials. In this study, sprouts were packaged in different permeabilities such as low-permeable (heat-sealed PVC film and LDPE bags), medium-permeable (LDPE film and micro-perforated LDPE bags), and highly permeable (macro-perforated LDPE bags).

After 10 days of storage, no significant differences were detected in weight loss in sprouts subjected to different packaging materials. On the contrary, after 28 days at 1 °C of storage, the weight loss of the sprout was much more accentuated in the macro-perforated LDPE bags compared to less permeable materials, with a consequent reduction of the shelf life to 21 days. A weight loss of −22.6% was detected after six days of storage at room temperature, whereas Li et al. [32] reported a weight loss of about −1.46% in bamboo sprouts packaged in sealed plastic vacuum materials after 15 days of storage at 25 °C. In this case, the vacuum lower weight loss of sprouts might be due to the subsequent vacuum packaging suppressing their moisture loss, as observed in our samples.

It is interesting to note that none of the packaging we have tested for *P. edulis* sprouts shows a weight loss of this magnitude even after 28 days of storage at 4 °C.

In the case of the sprout’s storage at 4 °C for 28 days, an a_w_ value of 0.957 and 0.950 for MAP1 and MAP2, was observed, respectively whereas a value of 0.948 was found for the vacuum-packaged sample. From the comparison of the pH values (Table S1), it is possible to observe that, independently, because of the packaging applied to the sprouts, a reduction in pH was observed during storage. The pH reaches its lowest value in sprouts packaged under vacuum after 28 days of storage at 4 °C (pH of 5.22).

As shown in Table 1, the firmness (FMN) of bamboo sprouts increased significantly during storage time, independently of the packaging conditions. This is probably due to the accumulation of cellulose and lignin in this portion, as reported in the literature [10,33]. No significant differences were recorded between sprouts packaged in MAP1 and MAP2. It is interesting to note that in sprouts, the basal section the firmness value of the vacuum-packaged sample is double compared to the initial one but at the same time lower than the samples packaged in MAP. Our data are in agreement with those found by Li et al. [32] as a consequence of the increase in lignin and fiber content during storage at 25 °C. Moreover, according to Pongprasert et al. [34] the accumulation of lignin during storage was limited in vacuum-packed samples. In our case, this evidence is limited to the basal section. A stable firmness value was observed in bamboo shoots packaged in linear low-density polyethylene (LLDPE) bags, with values in the sprout basal section ranging from 11.3 to 12.63 N after two and seven days of storage, respectively [35].

**Figure 2:**
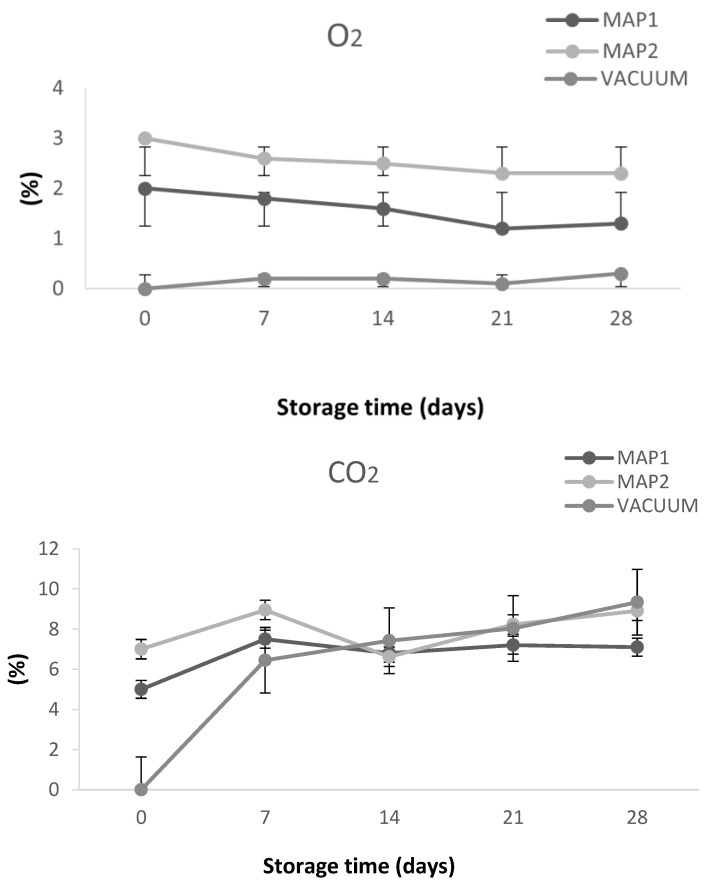
Headspace composition (%) in fresh peeled bamboo sprouts packaged in modified atmosphere packaging (MAP) and vacuum packaging systems. MAP1: 2% O_2_, 5% CO_2_, 93% N_2_; MAP2: 3% O_2_, 7% CO_2_, 90% N_2_.

The changes in headspace gas composition (O_2_ and CO_2_ concentration) under bamboo sprout packaging conditions are shown in Figure 2. Bamboo sprouts showed rapid and important changes in gas concentrations after packaging in MAP. In fact, according to Song et al. [36], the O_2_ concentration decreased moderately in this type of packaging in the first 7 days and then between 14 and 21 days of storage, with O_2_ concentrations ranging from 2 to 1.8% and from 3 to 2.6% for MAP1 and MAP2 at days 0 and 7, respectively. With these two types of packaging, the O_2_ concentration reached, at day 28, a percentage of 2.3 and 1.3 for MAP1 and MAP2 treatments, respectively. An increase in O_2_ concentration from 0 to 0.3% was observed in sprouts packaged in sealed vacuum bags. Regarding CO_2,_ an increase in its concentration was observed in all packaged samples. This increase is sharp in the first seven days of storage for vacuum-packaged samples (from 0 to 6.45%). A slight reduction in CO_2_ concentration was found in the samples packaged in MAP between the 7th and 14th days of storage, particularly for MAP2 packaged sprouts (from 8.95 to 6.62%). The evolution of headspace composition during storage of our samples agrees with Nugrahedi et al. [37], who observed a decreasing trend in O_2_ and an increasing trend in CO_2_. This condition is probably due to the respiration of the sprouts and the microbial growth.

The concentration of both O_2_ and CO_2_ inside the package changed during the storage period probably due to the process of respiration, which converts sugar into energy in the presence of oxygen, and the metabolism of microorganisms that consume O_2_ and produce CO_2_. Moreover, all operations that cause sprouts damage (cutting, transport, etc.) cause an increase in the respiration rate of fresh products. Other important factors are temperature of storage and packaging permeability. In fact, gas exchange between the external and internal atmospheres, mediated by the permeable polymer film, also influences the concentration of gases in the headspace.

For this reason, in order to prevent the loss of quality of sprouts as a consequence of the respiration process, it is critical to reduce the respiration rate as low as possible on the premise of maintaining essential living activities [38]. A stronger increase in CO_2_ and a decrease in O_2_ are necessary. This condition is possible by using packaging materials that by less permeable to these gas [39].

### 3.2. Effect of Storage on Packaged Bamboo Sprouts CIELab Parameters

The food color is an important quality parameter, as it influences consumers’ purchasing choices. To evaluate the impact of the packaging systems on the fresh sprouts without sheath, it was decided to divide them into two sections: high (apical) and low (basal), and to measure the CIELab parameters on these two sections separately during the sample monitoring period (28 days). As can be seen in Table S2, there is a significant difference between vacuum packaging and modified atmosphere packaging.

Generally, a reduction in both the C* and L* parameters was observed during storage regardless of the packaging system applied for preserving fresh sprouts packaged in MAP (Table 2 and Table 3). The reduction of the L* parameter appears more pronounced in the basal part of the sprout, with values ranging from 59.72 to 46.22 for sprouts packaged in the MAP2 system. A similar trend was also observed for the C* parameter. No significant differences in L* were observed in vacuum-packaged sprouts during storage.

The discoloration process that is linked to L* and C* parameters occurred due to phenylalanine ammonia lyase and peroxidase enzymatic activity that were activated by tissue injury due to postharvest [30]. At the same time Shen et al. [40] demonstrated that the application of MAP allows the sprouts to remain edible despite the slight browning. According to our data, Changchai et al. [35], who investigated the impact of storage conditions on bamboo shoots packaged in LLDPE bags, found a significant reduction in the L* parameter of the sprout basal portion from 2 to 7 days of storage at 5 ± 3 °C. At the same time, the use of LLDPE bags did not determine a reduction in C* parameters as observed in our case in the sprout basal section. A decrease in L* parameters was also observed by Li et al. [32] on blanched bamboo sprouts packaged in vacuum-sealed plastic vacuum packaging materials. In this case, the application of high hydrostatic pressure (300 MPa for 10 min) produced a higher overall color. A similar observation was made also in bamboo sprouts packaged in PE bags and stored for 60 days at 2 °C with L* values ranging from 78.99 to 65.38 [41].

As it is well known, browning is very common in the processing and storage of fruits and vegetables. For this reason, the effectiveness of treatments was evaluated in terms of color variations. The total color difference (ΔE) is a parameter used to indicate the difference in color in food following its processing or storage.

The ΔE parameter has shown significant differences since day 0 and became significant at t = 28 (Table S3). This variability is also found when calculating the BI (Table 4). BI is an important parameter indicating color variations because of enzymatic and non-enzymatic reactions.

Observing data relating to the Browning Index (BI) (Table 4), a different behaviour emerges between the two sections, further supporting our operational choice to divide the sprouts into two sections to carry out monitoring. In general, a reduction in BI was observed in samples packaged in modified atmosphere packaging, whereas no significant differences were observed between apical and basal sections in vacuum-packaged samples.

A slight increase in BI was observed during the storage of *P. praecox* sprouts packaged in fibreboard cartons and stored at 1 °C; within 20 days of storage there was a quick increase in BI [42].

### 3.3. Microbiological Determination

Bacteria, yeasts, and molds, in addition to being typical and characterizing constituents of many food products, in some cases represent a danger to the safety and integrity of foods. During food storage, it is necessary to guarantee that there are no microbial growths that could alter the product, which would cause a potential danger for the consumer. Each food is associated with a different microbiological risk, which means that for each product, it is necessary to evaluate which microorganisms can constitute an actual danger and how to monitor them during the tests.

These assessments must be carried out during the preliminary study phase, in which all aspects connected with the presence and possibility of the development of microorganisms will be taken into consideration, including the ingredients and additives used, the processing methods, the packaging, storage temperatures, and the chemical-physical characteristics of the food, such as pH and free water values. From the comparison of the three packaging systems, significantly different data was observed (Table 5).

A more marked qualitative deterioration from a microbiological point of view is evidenced in the sample packaged in MAP1. This condition is particularly evident at 28 days of storage with a TBC of 9.60 log_10_ UFC/g vs. 4.77 log_10_ UFC/g (at day 0). A similar situation was observed with yeast. This data is unexpected since this packaging system has the lowest percentage of oxygen, which is necessary for the growth of numerous microorganisms. No molds were detected in all the investigated samples. An increase in TBC was observed, similar to findings of Li et al. [32], where bamboo sprouts packaged in vacuum material showed a TBC of 1.04 × 10^2^ CFU/g on day 0 and 3.74 × 10^2^ CFU/g after 15 days of storage at 25 °C. Previously, Kleinhenz et al. [10] evidenced a different bacterial and mycological count in *B. oldhamii* between the external sheath and internal tissue.

### 3.4. Analysis of Selected Organic Acids

Bamboo sprouts easily undergoes a fermentation process, which is why it was necessary to observe the evolution of the most common organic acids present in the matrix. The qualitative-quantitative profile of some sprout markers was monitored, including oxalic, tartaric, malic, citric, succinic, and fumaric acid (Table 6). Except for fumaric acid, the content of all markers undergoes a reduction over time. Previously, Chen et al. [42] demonstrated that malic, lactic, and tartaric acids were the main organic acids in sour *Dendrocalamus giganteus* sprouts.

### 3.5. Evolution of Bioactive Compounds (TPC, TFC, and TCC) in Bamboo Sprouts during Storage

The study continued in terms of the evaluation of the content of bioactive compounds and of total phenol (TPC), flavonoid (TFC), and carotenoid (TCC) through spectrophotometric assays. Fresh bamboo sprouts extract at day 0 of storage showed a TPC of 85.42 mg of chlorogenic acid equivalents (CAE)/g of fresh sample. In the case of TPC, the trend in the samples packaged in MAP can be understood as fluctuating, with a reduction in the first seven days of storage and then an increase observed after 28 days of storage at 4 °C. On the contrary, in vacuum packaging a time-dependent decline in the quantity of these bioactive compounds is observed. A similar trend is observed in the case of flavonoids and carotenoids. This could be related to the lack of oxygen in the vacuum packaging, which requires the functioning of enzymes involved in metabolic processes involving these phytochemicals, including TPC, TFC, and TCC (Table 7).

Analysis of the literature reported a TPC content between 101.65 and 721.62 mg/100 g of sample for *B. balcooa* and *B. bambos*, respectively [43,44].

The TPC of our sample is higher than that reported by Shen et al. for *P. heterocycle* (1.6 mg CAE/g of fresh weight) [42]. The great variability in the TPC found in the literature may depend on the fact that the presence of these phytochemicals differs between the apical and basal portions of the sprout. In fact, in the case of *P. heterocycla* var. *pubescens*, a maximum content was found in the apical region of the sprout (about 60 mg CAE/g of fresh weight) compared to the middle and basal portions [45].

The content of TPC, and consequently, of TFC depends on the sprout’s maturity stage. In fact, both phytochemicals tend to reduce as the sprouts mature due to enzymatic reactions and oxidative processes, as well as during storage [46,47,48,49], which investigated the evolution of TPC in *P. violascens* sprouts and found the maximum value at day 0 (23.50 mg CAE/g of fresh weight).

A decrease in the phenolic content of packaged sprouts was observed compared to sprouts at the beginning of storage (day 0). This decrease in phenolic content could be related to sprouts respiration during storage. The results showed that the vacuum-packaged sprouts had a higher TPC content after 28 days of storage. The trend observed during storage is probably due to the activity of polyphenol oxidase (PPO), the enzyme responsible for the oxidation of phenols, and this increased activity could be responsible for the decrease in phenolic content during storage. Several studies have shown that the less oxygen stored in the atmosphere of a vegetable, the greater its phenol content, since a high oxygen content causes the oxidation of phenols (through greater PPO activity) and reduces its total phenolic content [50,51].

### 3.6. Degradation Kinetics of TPC, TFC, and TCC during Storage

Kinetic modeling is currently applied to explain and predict the change in product quality because of different packaging conditions. In our case, the degradation kinetics of quantified bioactive compounds (TPC, TFC, and TCC) was performed.

The data shows that all the parameters being analysed follow a zero-order reaction (Table 8). The results indicate that the modified atmosphere storage treatment causes a more rapid decrease in TPC, which is in line with results from previous studies on the degradation of polyphenols preserved with different packaging techniques [50,51,52]. During storage, the observed phytochemicals reactions (TPC and TFC) are probably due to the activity of polyphenol oxidase (PPO), the enzyme responsible for the oxidation of phenols, and this increased activity could be responsible for the decrease in phenolic content during storage. In fact, the half-life of total polyphenols is greater in bamboo sprouts preserved under vacuum than in a modified atmosphere.

The degradation of TPC followed zero-order kinetics, as previously described. The reaction rate constants [k = −ln(C_t_/C_0_*t)] and the half-life values [t_1/2_ = ln(2)/k] of the degradation showed that the reaction was the slowest in the case of vacuum (k = 1.3270 day^−1^, t_1/2_ = 0.5223 days), followed by MAP1 (k = 1.4071 day^−1^, t_1/2_ = 0.4927 days) and MAP2 (k = 1.4960 day^−1^, t_1/2_ = 0.4634 days). A similar trend was also observed in TFC and TCC degradation. In both cases, phytochemicals present in a sample stored in a vacuum undergo a more pronounced degradation process.

### 3.7. UHPLC Analysis of Fresh Bamboo Sprouts

Selected phytochemicals, mainly phenolic acids, were identified and quantified in bamboo sprout extract (Figure S1). Among the phenolic acids, protocatechuic acid (636.91 µg/g), and caffeic acid (455.81 µg/g) were the most abundant, followed by gallic acid (156.03 µg/g) and chlorogenic acid (145.12 µg/g). *p*-Coumaric and ferulic acids were less abundant (74.90 and 81.22 µg/g). Isoorientin (364.67 µg/g) was the main flavonoid identified in sprout extract, followed by isovitexin (135.13 µg/g). Rutin (139.41 µg/g) was also found.

Our data are in agreement with those found in other fresh sprouts, where ferulic acid, *p*-coumaric acid, caffeic acid, protocatechuic acid, *p*-hydroxybenzoic acid, catechin, syringic acid, and chlorogenic acid were the main abundant compounds [53]. Previously, Park and Jhon [54] compared the phenolic acid composition of *P. nigra* sprouts subjected to extraction procedures with different solvents and demonstrated that, for example, chlorogenic acid should be extracted with EtOAc and BuOH with values of 4.1 and 3.5 mg/100 g dry weight, but not with MeOH and water. Gallic acid, ferulic acid, and protocatechuic acid were also identified in the *P. edulis* shoot tip by Li et al. [55]. Moreover, no significant differences were evidenced when comparing the identified phenolic acid and flavonoid content in our samples and the sheath and leaves of *P. edulis* collected in the same area [27].

### 3.8. Investigation on the Evolution of Bamboo Sprouts Antioxidant Activity during Storage

The deterioration of food matrix due to oxidation involves both the aqueous phase (e.g., proteins) and the lipid phase (e.g., polyunsaturated lipids). The formation of free radicals is the first event, followed by the progression of oxidation, and is most often associated with the aqueous phase. The possibility of protecting foods from the oxidative deterioration of their vulnerable constituents is today a major challenge, especially if it is currently obtainable in the absence of the addition of substances with the function of antioxidant additives.

The antioxidant activity of fresh bamboo samples packaged under different packaging systems was investigated, and the results are reported in Table 9.

Generally, samples exhibited the most promising radical scavenging activity against ABTS^+^ in comparison with DPPH (Table 9) with IC_50_ values in the range of 0.78–2.27 μg/mL, 0.78–3.56 μg/mL, and 0.78–4.41 μg/mL for MAP1, MAP2, and vacuum-packaged, respectively.

It is interesting to note that in the DPPH test, the IC_50_ value at 28 days of storage was approximately two times higher in the vacuum sample in comparison with MAP-packaged samples. No significant differences were recorded in our samples in the β-carotene bleaching test, used to investigate the protection against lipid peroxidation, where, after 60 min of incubation, IC_50_ values of 14.29, 16.89, and 19.31 μg/mL were found after 28 days of storage for MAP1, MAP2, and vacuum packages, respectively. The fresh bamboo sprout extracts did not exhibit ferric-reducing ability. In general, a great variability in antioxidant potential was observed in the samples, regardless of the packaging applied.

This does not allow us to identify a clear trend to identify the best preservation method capable of preserving the antioxidant power of the sprouts. This situation is probably linked to the fact that the antioxidant activity is not related to a single compound or class of compounds but is the result of synergism between the different phytochemicals that characterize the extract [56].

Recent studies have evidenced that the application of food processing to bamboo sprouts can modify their health properties, including their antioxidant potential, probably due to the effect of these processes on the content of bioactive compounds [57,58,59]. Santosh et al. [60] investigated the DPPH inihibitory activity of fresh Dendrocalamus hamiltonii sprouts and found an IC_50_ value of 2824.24 μg/mL. The antioxidant properties of Giganthochla psedoarundinacea, Dendrocalamus asper, Giganthochla apus, and Bambusa vulgaris var. striata bamboo sprout extracts were evaluated by a DPPH radical assay by Iwansyah et al. [61], which found IC_50_ values in the range 347.48–2489.60 μg/mL. All these values are higher than those found on our fresh samples, even after 28 days of storage. On the contrary, an IC_50_ value of 9.20 μg/mL was registered using P. pubescens sprouts subjected to a microwave-assisted extraction process [62]. Previously, Badwaik et al. [43] demonstrated that DPPH radical scavenging potential was influenced by the bamboo sprout fermentation process. In fact, percentages of DPPH radical scavenging activity of 26.62, 49.20, and 55.35% were found for fresh and fermented sprouts by natural anaerobic fermentation or fermentation in the presence of pieces of Garcinia pedunculata, respectively.

Pearson’s correlation coefficient revealed a positive correlation between TPC and DPPH for all the observed days of storage, independently of the applied treatment, with r values ranging from 0.99 to 1 after 7 and 21 days of storage, respectively. Moreover, TPC is positively correlated with protection from lipid peroxidation, independently of the time of incubation (30 and 60 min), with values ranging from 0.57 to 0.99 after 7 and 28 days of storage, respectively, and from 0.80 to 0.94 after 21 and 28 days of storage, respectively. This evidence means that TPC is strictly involved in the bioactivity of bamboo sprouts. This trend was also confirmed by TFC correlation values.

### 3.9. Principal Component Analysis (PCA)

Principal Component Analysis (PCA) was performed to identify accession groups and determine the axes and characters significantly contributing to the variation. In this procedure, the similarity matrix was used to generate eigenvalues and scores for the accessions. PCA was applied to differentiate the three different treatments (MAP1, MAP2, and VACUUM).

**Figure 3:**
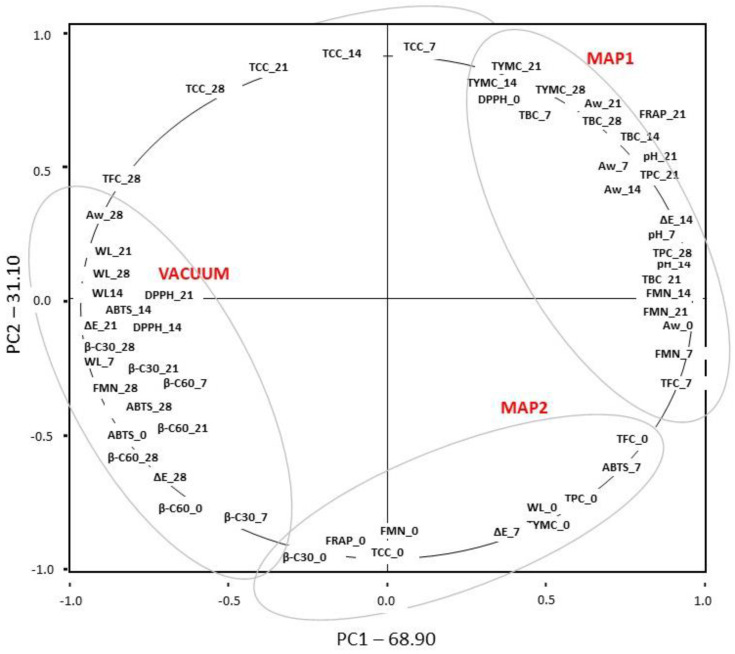
Principal component analysis of the physico-chemical attributes of shelf-life fresh bamboo sprouts packaged in a modified atmosphere and vacuum. PCA of the variables showing their major contribution and PCA-biplot analysis representing the performance of quality parameters. MAP: modified atmosphere packaging. MAP1: 2% O_2_, 5% CO_2_, 93% N_2_; MAP2: 3% O_2_, 7% CO_2_, 90% N_2_; WL: weight loss; FMN: firmness; ΔE: distance between two colors in a color space; TBC: Total bacterial count; TYMC: total yeast and mold count; TPC: total phenol content; TFC: total flavonoid content; TCC: total carotenoid content; DPPH: 2,2-diphenyl-1-picrylhydrazyl; ABTS: 2,2′-azino-bis (3-ethylbenzothiazoline-6-sulfonic acid; Bcar/30: β-carotene bleaching 30 min incubation; Bcar/60: β-carotene bleaching 60 min incubation; FRAP: ferric reducing antioxidant power.

By choosing eigenvalues greater than one (>1), the dimensionality was reduced from 80 variables to two principal components (PC). The results were analyzed by a multivariate PCA method to reach a smaller number of artificial variables accounting for most of the variance in the observed variables [63]. The obtained results are shown in Figure 3. In all considered samples, the first two PCs accounted for 100% of the total variance.

The Scree plot shows the variance of each component in the dataset, used to determine how many components should be retained in order to explain a high percentage of the variation in the data. As can be seen in Figure 3, the variance is explained by PC1 and PC2. In particular, PC1 explains about 68.90% and PC2 about 31.10% (Table S4) of the total variance (100%). The analysis of the graphs underlined possible differences and analogies among varieties and evaluated variables. The first component (PC1) is positively correlated with FMN_7, FMN_14, FMN_21, Aw_0, Aw_7, Aw_14, Aw_21, pH_0, pH_7, pH_14, pH_21, pH_28, ΔE_14, TBC_0, TBC_7, TBC_14, TBC_21, TBC_28, TYMC_7, TYMC_14, TYMC_21, TYMC_28, TPC_0, TPC_7, TPC_14, TPC_21, TPC_28, TFC_0, TFC_7, TFC_14, TFC_21, DPPH_0, ABTS_7, FRAP_7, FRAP_14, FRAP_21, FRAP_28.

The second component (PC2) is positively correlated with TYMC_7, TYMC_14, TYMC_21, TYMC_28, TCC_7, TCC_14, TCC_21, TCC_28, DPPH_0, FRAP_7, FRAP_14, FRAP_21, and FRAP_28.

The scores plot analysis clearly classifies the treatments in the regions of the PCA score plot (first, second, and third quadrant). The analysis demonstrated that the treatment analyzed, MAP1, was located in the top right quadrant, which represents the highest TPC_21, TPC_28, DPPH_0, and FRAP_21. The MAP2 treatment was located in the lower right quadrant, which represents the highest TPC_0, TFC_0, and ABTS_7. The vacuum treatment was located in the top left quadrant, which represents the highest levels of TFC_28, TCC_7, TCC_14, TCC_21, and TCC_28.

## 4. Conclusions

In this study, the impact of vacuum and atmosphere packaging systems on *Phyllostacys edulis* sprout quality parameters, bioactive compound content, and antioxidant activity were evaluated. Our results confirm the superiority of MAP compared to vacuum packing, regardless of the gas mixture placed inside the packaging.

By evaluating the half-life of the bioactive compounds (TPC, TFC, and TCC) as well as the antioxidant activity of the bamboo sprouts, the superiority of MAP1 packaging systems, characterized by the following gas mixture, 2% O_2_, 5% CO_2_, and 93% N_2_, emerges as confirmed by PCA analysis.

Further studies should be performed to test our MAP1 packaging system combined with different preventive treatments of the sprouts with decontaminants (i.e., salicylic acid), NO, ozone, γ-irradiation, or high hydrostatic pressure, but always at a low storage temperature since this temperature allows to maintain a constant respiration rate.

## Figures and Tables

**Table 1:** Firmness parameter value (N) in sprouts packaged in MAP and vacuum: (a) apical, (b) basal sections.

Storage Time (Days)	MAP1	MAP2	VACUUM	
		Apical section		Sign.
0	8.68 ± 0.87 ^aA^	8.68 ± 0.87 ^aA^	8.68 ± 0.87 ^aA^	ns
7	9.79 ± 1.44 ^bA^	9.62 ± 1.67 ^bA^	8.89 ± 0.67 ^bB^	**
14	10.16 ± 2.43 ^cA^	10.16 ± 1.33 ^cA^	9.22 ± 0.89 ^cB^	**
21	10.82 ± 0.88 ^dA^	10.8 ± 1.08 ^bA^	9.60 ± 0.78 ^dB^	**
28	16.72 ± 1.22 ^eA^	16.79 ± 1.78 ^eA^	16.72 ± 1.43 ^eA^	**
Sign.	**	**	**	
		Basal section		Sign.
0	8.53 ± 0.56 ^aA^	8.53 ± 0.56 ^aA^	8.53 ± 0.56 ^eA^	ns
7	10.71 ± 1.67 ^bA^	10.71 ± 2.02 ^bA^	8.71 ± 1.78 ^bB^	**
14	11.99 ± 0.99 ^bA^	11.99 ± 1.41 ^bA^	8.98 ± 1.45 ^cB^	**
21	12.19 ± 2.31 ^cA^	12.09 ± 1.78 ^cA^	10.14 ± 1.69 ^dB^	**
28	29.03 ± 1.56 ^dA^	29.03 ± 2.67 ^dA^	16.43 ± 0.435 ^dB^	**
Sign.	**	**	**	

MAP: modified atmosphere packaging. MAP1: 2% O_2_, 5% CO_2_, 93% N_2_; MAP2: 3% O_2_, 7% CO_2_, 90% N_2_. Data are reported as mean ± standard deviation (n = 3). Results followed by different capital letters in the same row indicate differences among the samples. The different lowercase letters in the same column indicate differences during storage. Differences within and between groups were evaluated by one-way ANOVA followed by Tukey’s test (**, *p* < 0.01). Results followed by different letters are significantly different with ** indicating a *p* ≤ 0.01, and ns indicating a *p* > 0.05 which is not significant.

**Table 2:** Lightness L* evolution in sprouts packaged in MAP and VACUUM during storage for 28 days at 4 °C.

Storage Time (Days)	MAP1	MAP2	VACUUM	Sign.
	Apical section			
0	59.22 ± 3.89 ^^aA^^	59.22 ± 2.78 ^^aA^^	20.62 ± 2.08 ^bB^	**
7	52.29 ± 4.76 ^cA^	51.39 ± 4.02 ^cB^	20.63 ± 2.03 ^abC^	**
14	51.02 ± 3.66 ^dA^	51.01 ± 3.61 ^dA^	20.62 ± 2.89 ^bB^	**
21	47.31 ± 3.67 ^eA^	47.12 ± 4.09 ^eB^	20.63 ± 2.99 ^bC^	**
28	53.82 ± 5.01 ^bA^	52.84 ± 4.67 ^bB^	20.65 ± 3.09 ^aC^	**
Sign.	**	**	*	
	Basal section			Sign.
0	59.72 ± 4.87 ^aA^	59.72 ± 4.74 ^aA^	20.65 ± 2.78 ^aB^	**
7	42.12 ± 3.81 ^eA^	41.12 ± 4.04 ^eB^	20.66 ± 1.98 ^aC^	**
14	54.82 ± 2.89 ^bA^	53.82 ± 4.06 ^bB^	20.64 ± 2.84 ^aC^	**
21	50.12 ± 2.88 ^cA^	49.12 ± 4.88 ^cB^	20.67 ± 2.77 ^aC^	**
28	48.22 ± 3.66 ^dA^	46.22 ± 4.07 ^dB^	20.65 ± 3.89 ^aC^	**
Sign.	**	**	ns	

MAP: modified atmosphere packaging. MAP1: 2% O_2_, 5% CO_2_, 93% N_2_; MAP2: 3% O_2_, 7% CO_2_, 90% N_2_. Data are reported as mean ± standard deviation (n = 3). Results followed by different capital letters in the same row indicate differences among the samples. The different lowercase letters in the same column indicate differences during storage. Differences within and between groups were evaluated by a one-way ANOVA followed by Tukey’s test (**, *p* < 0.01). Results followed by different letters are significantly different with * indicating a *p* ≤ 0.05, ** indicating a *p* ≤ 0.01, and ns indicating a *p* > 0.05 which is not significant.

**Table 3:** Chroma C* in sprouts packaged in MAP and VACUUM during storage for 28 days at 4 °C.

Storage Time (Days)	MAP1	MAP2	VACUUM	
	Apical section			Sign.
0	19.43 ± 1.21 ^aA^	19.43 ± 1.12 ^aA^	1.59 ± 0.02 ^aB^	**
7	2.45 ± 0.03 ^dA^	2.36 ± 0.06 ^cB^	1.17 ± 0.01 ^cC^	**
14	2.32 ± 0.02 ^eA^	2.25 ± 0.04 ^cB^	1.38 ± 0.01 ^bC^	**
21	2.76 ± 0.06 ^cA^	2.72 ± 0.03 ^bA^	1.17 ± 0.01 ^cB^	**
28	7.75 ± 0.11 ^bA^	2.70 ± 0.03 ^bB^	1.59 ± 0.01 ^aC^	**
Sign.	**	**	**	
	Basal section			Sign.
0	19.98 ± 2.12 ^aA^	19.98 ± 1.97 ^aA^	1.17 ± 0.02 ^bB^	**
7	2.1 ± 0.03 ^eA^	2.10 ± 0.03 ^eA^	1.17 ± 0.01 ^bB^	**
14	3.81 ± 0.07 ^bA^	3.67 ± 0.05 ^bB^	1.59 ± 0.04 ^aC^	**
21	3.05 ± 0.05 ^cA^	3.05 ± 0.04 ^cA^	1.18 ± 0.02 ^bB^	**
28	2.61 ± 0.07 ^dA^	2.58 ± 0.04 ^dA^	1.58 ± 0.02 ^aB^	**
Sign.	**	**	**	

MAP: modified atmosphere packaging. MAP1: 2% O_2_, 5% CO_2_, 93% N_2_; MAP2: 3% O_2_, 7% CO_2_, 90% N_2_. Data are reported as mean ± standard deviation (n = 3). Results followed by different capital letters in the same row indicate differences among the samples. The different lowercase letters in the same column indicate differences during storage. Differences within and between groups were evaluated by a one-way ANOVA followed by Tukey’s test (**, *p* < 0.01). Results followed by different letters are significantly different with ** indicating a *p* ≤ 0.01, and ns indicating a *p* > 0.05 which is not significant.

**Table 4:** Browning Index parameter evolution during storage for bamboo sprouts packaged in MAP and vacuum.

Storage Time (Days)	MAP1	MAP2	VACUUM	Sign.
	Basal section			
0	43.52 ± 4.98 ^aA^	43.52 ± 4.76 ^aA^	11.76 ± 1.78 ^aB^	**
7	11.76 ± 1.67 ^dA^	11.77 ± 1.54 ^dA^	11.77 ± 1.45 ^aA^	ns
14	29.41 ± 2.78 ^bA^	29.18 ± 3.03 ^bB^	11.76 ± 3.44 ^aC^	**
21	17.65 ± 1.04 ^cA^	17.65 ± 2.33 ^cA^	11.77 ± 2.62 ^aB^	**
28	17.64 ± 1.38 ^cA^	11.77 ± 1.44 ^dB^	11.76 ± 0.37 ^aB^	**
Sign.	**	**	ns	
	Apical section			Sign.
0	42.36 ± 3.44 ^aA^	42.36 ± 3.83 ^aA^	11.76 ± 1.43 ^aB^	**
7	11.77 ± 1.23 ^cA^	11.78 ± 1.32 ^cA^	5.88 ± 0.57 ^aB^	**
14	11.76 ± 0.93 ^cA^	11.79 ± 0.89 ^cA^	11.76 ± 0.49 ^aA^	ns
21	11.65 ± 0.54 ^bA^	11.65 ± 0.67 ^bA^	11.76 ± 0.64 ^aB^	**
28	11.77 ± 0.87 ^cA^	11.77 ± 0.97 ^cA^	11.76 ± 0.85 ^aA^	ns
Sign.	**	**	ns	

MAP: modified atmosphere packaging. MAP1: 2% O_2_, 5% CO_2_, 93% N_2_; MAP2: 3% O_2_, 7% CO_2_, 90% N_2_. Data are reported as mean ± standard deviation (n = 3). Results followed by different capital letters in the same row indicate differences among the samples. The different lowercase letters in the same column indicate differences during storage. Differences within and between groups were evaluated by a one-way ANOVA followed by Tukey’s test (**, *p* < 0.01). Results followed by different letters are significantly different with ** indicating a *p* ≤ 0.01, and ns indicating a *p* > 0.05 which is not significant.

**Table 5:** Total bacterial count (TBC) and total yeast and mold count (TYMC) (log_10_ UFC/g) in bamboo sprouts subjected to different packaging systems.

TBC	MAP1	MAP2	VACUUM	Sign.
0	4.77 ± 0.20 ^aC^	4.77 ± 0.20 ^aD^	4.47 ± 0.20 ^bD^	**
7	4.30 ± 0.29 ^aD^	3.90 ± 0.29 ^bE^	3.86 ± 0.29 ^bE^	**
14	6.80 ± 0.09 ^aB^	6.40 ± 0.05 ^bC^	6.10 ± 0.05 ^cC^	**
21	6.96 ± 0.08 ^aB^	6.86 ± 0.04 ^bB^	6.26 ± 0.04 ^cB^	**
28	9.60 ± 0.56 ^aA^	9.00 ± 0.46 ^bA^	8.65 ± 0.46 ^cA^	**
Sign.	**	**	**	
TYMC	MAP1	MAP2	VACUUM	
0	nd	nd	nd	ns
7	1.81 ± 0.28 ^aD^	1.71 ± 0.28 ^bD^	1.70 ± 0.28 ^bD^	**
14	3.00 ± 0.02 ^aC^	2.20 ± 0.02 ^bC^	2.18 ± 0.02 ^bC^	**
21	3.86 ± 0.41 ^aB^	3.16 ± 0.41 ^bB^	3.10 ± 0.41 ^bB^	**
28	4.90 ± 0.06 ^aA^	3.90 ± 0.06 ^bA^	3.80 ± 0.06 ^bA^	**
Sign.	**	**	**	

MAP: modified atmosphere packaging. MAP1: 2% O_2_, 5% CO_2_, 93% N_2_; MAP2: 3% O_2_, 7% CO_2_, 90% N_2_. Data are reported as mean ± standard deviation (n = 3). Nd: not detected. Results followed by different capital letters in the same row indicate differences among the samples. The different lowercase letters in the same column indicate differences during storage. Differences within and between groups were evaluated by a one-way ANOVA followed by Tukey’s test (**, *p* < 0.01). Results followed by different letters are significantly different with ** indicating a *p* ≤ 0.01, and ns indicating a *p* > 0.05 which is not significant.

**Table 6:** Analysis of selected acid markers in bamboo sprouts subjected to different packaging systems.

	Ossalic Acid	Tartaric Acid	Malic Acid	Citric Acid	Succinic Acid	Fumaric Acid
MAP1						
0	323.84 ± 0.20 ^a^	145.14 ± 0.35 ^b^	79.6 ± 0.31 ^a^	307.09 ± 0.26 ^a^	133.76 ± 1.00 ^a^	0.6 ± 0.00 ^e^
7	273.04 ± 0.27 ^b^	224.96 ± 0.31 ^a^	nd	76.33 ± 0.46 ^c^	nd	1.26 ± 0.00 ^c^
14	115.82 ± 0.22 ^d^	nd	nd	36.04 ± 1.22 ^c^	nd	0.65 ± 0.00 ^d^
21	242.99 ± 0.09 ^e^	14.94 ± 0.21 ^d^	nd	93.6 ± 0.27 ^b^	nd	1.66 ± 0.00 ^b^
28	246.04 ± 0.11 ^c^	53.93 ± 0.10 ^c^	nd	72.01 ± 0.27 ^c^	nd	1.92 ± 0.00 ^a^
Sign.	**	**	**	**	**	**
MAP2						
0	323.84 ± 0.20 ^a^	145.14 ± 0.35 ^b^	79.6 ± 0.31 ^a^	307.09 ± 0.26 ^a^	133.76 ± 1.00 ^a^	0.6 ± 0.00 ^e^
7	268.04 ± 0.27 ^b^	212.96 ± 0.31 ^a^	nd	75.33 ± 0.46 ^c^	nd	1.23 ± 0.00 ^c^
14	120.82 ± 0.22 ^d^	nd	nd	32.04 ± 1.22 ^c^	nd	0.63 ± 0.00 ^d^
21	222.89 ± 0.09 ^e^	12.34 ± 0.21 ^d^	nd	85.36 ± 0.27 ^b^	nd	1.61 ± 0.00 ^b^
28	236.44 ± 0.11 ^c^	49.83 ± 0.10 ^c^	nd	70.11 ± 0.27 ^c^	nd	1.90 ± 0.00 ^a^
Sign.	**	**	**	**	**	**
VACUUM						
0	321.52 ± 0.20 ^a^	144.14 ± 0.35 ^b^	79.6 ± 0.31 ^a^	306.19 ± 0.26 ^a^	133.76 ± 1.00 ^a^	0.6 ± 0.00 ^e^
7	266.04 ± 0.27 ^b^	209.96 ± 0.31 ^a^	nd	75.03 ± 0.46 ^c^	nd	1.02 ± 0.00 ^c^
14	118.12 ± 0.22 ^d^	nd	nd	31.84 ± 1.22 ^c^	nd	0.61 ± 0.00 ^d^
21	202.88 ± 0.09 ^e^	12.04 ± 0.21 ^d^	nd	84.96 ± 0.27 ^b^	nd	1.58 ± 0.00 ^b^
28	234.41 ± 0.11 ^c^	48.83 ± 0.10 ^c^	nd	68.21 ± 0.27 ^c^	nd	1.86 ± 0.00 ^b^
Sign.	**	**	**	**	**	**

MAP: modified atmosphere packaging. MAP1: 2% O_2_, 5% CO_2_, 93% N_2_; MAP2: 3% O_2_, 7% CO_2_, 90% N_2_. Data are reported as mean ± standard deviation (n = 3). Results followed by different capital letters in the same row indicate differences among the samples. The different lowercase letters in the same column indicate differences during storage. Differences within and between groups were evaluated by a one-way ANOVA followed by Tukey’s test (**, *p* < 0.01). Results followed by different letters are significantly different with ** indicating a *p* ≤ 0.01, and ns indicating a *p* > 0.05 which is not significant.

**Table 7:** Evolution of bioactive content in sprouts packaged in MAP and vacuum during storage for 28 days at 4 °C.

Total Phenol Content (TPC)				
Storage Time (Days)	MAP 1	MAP 2	VACUUM	Sign
0	85.42 ± 4.1 ^aA^	85.42 ± 4.1 ^aA^	85.42 ± 4.1 ^aA^	ns
7	66.34 ± 5.3 ^bA^	66.30 ± 5.5 ^bA^	67.11 ± 5.8 ^bA^	**
14	66.87 ± 4.4 ^bA^	66.80 ± 4.8 ^bA^	66.94 ± 4.7 ^bA^	**
21	52.08 ± 5.2 ^cA^	52.00 ± 5.5 ^cA^	54.53 ± 5.0 ^CA^	**
28	43.30 ± 5.0 ^dA^	40.21 ± 5.6 ^dB^	45.24 ± 5.3 ^dC^	**
Sign.	**	**	**	
Total flavonoid content (TFC)				
Storage time (days)	MAP 1	MAP 2	VACUUM	Sign.
0	46.97 ± 4.6 ^aA^	46.97 ± 4.6 ^aA^	46.97 ± 4.6 ^aA^	ns
7	36.74 ± 4.3 ^bA^	38.88 ± 4.4 ^bB^	31.32 ± 3.4 ^bC^	**
14	36.30 ± 3.3 ^bA^	35.14 ± 3.7 ^cB^	26.75 ± 4.2 ^cC^	**
21	28.60 ± 4.1 ^cA^	27.50 ± 4.0 ^dB^	25.87 ± 4.0 ^dC^	**
28	20.31 ± 5.5 ^dA^	18.88 ± 3.9 ^eA^	23.07 ± 4.1 ^eB^	*
Sign.	**	**	**	
Total carotenoid content (TCC)				
Storage time (days)	MAP 1	MAP 2	VACUUM	Sign.
0	29.80 ± 4.2 ^aA^	29.80 ± 4.2 ^aA^	29.80 ± 4.2 ^aB^	ns
7	25.65 ± 4.1 ^bA^	23.46 ± 4.0 ^bB^	24.52 ± 3.8 ^cC^	**
14	23.38 ± 3.8 ^bA^	21.22 ± 3.7 ^cB^	22.92 ± 3.4 ^cC^	**
21	23.20 ± 3.7 ^bA^	21.38 ± 3.6 ^cA^	23.44 ± 3.2 ^dB^	**
28	18.20 ± 1.5 ^dA^	16.60 ± 3.2 ^dA^	18.86 ± 2.7 ^bB^	**
Sign.	**	**	**	

MAP: modified atmosphere packaging. MAP1: 2% O_2_, 5% CO_2_, 93% N_2_; MAP2: 3% O_2_, 7% CO_2_, 90% N_2_. Data are reported as mean ± standard deviation (n = 3). Results followed by different capital letters in the same row indicate differences among the samples. The different lowercase letters in the same column indicate differences during storage. Differences within and between groups were evaluated by a one-way ANOVA followed by Tukey’s test (**, *p* < 0.01). Results followed by different letters are significantly different with * indicating a *p* ≤ 0.05, ** indicating a *p* ≤ 0.01, and ns indicating a *p* > 0.05 which is not significant.

**Table 8:** First-order degradation kinetic parameters of TPC, TFC, and TCC in bamboo sprouts subjected to different packaging systems and stored at 4 °C for 28 days.

Bioactives	Packaging Systems	Reaction Order	*K*-Value	Equation	R^2^	Half-Life (t_1/2_) (Days)
TPC						
	MAP1	0	1.4071	y = −1.4071x + 82.502 R^2^ = 0.9366	0.9447	0.4927
	MAP2	0	1.4960	y = −1.496x + 83.09 R^2^ = 0.9415	0.9415	0.4634
	VACUUM	0	1.3270	y = −1.327x + 82.436 R^2^ = 0.9403	0.9403	0.5223
TFC						
	MAP1	0	0.8780	y = −0.878x + 46.076 R^2^ = 0.9506	0.9506	0.7894
	MAP2	0	0.9651	y = −0.9651x + 46.986 R^2^ = 0.9862	0.9862	0.7182
	VACUUM	0	0.7607	y = −0.7607x + 41.446 R^2^ = 0.7829	0.7829	0.9112
TCC						
	MAP1	0	2.5650	Y = −2.565x + 31.741 R^2^ = 0.9264	0.9264	0.2702
	MAP2	0	2.8480	y = −2.848x + 31.036 R^2^ = 0.88825	0.8825	0.2433
	VACUUM	0	2.2960	y = −2.296x + 3.4515 R^2^ = 0.8535	0.8535	0.3019

Total phenol content (TPC); total flavonoid content (TFC); total carotenoid content (TCC). MAP: modified atmosphere packaging. MAP1: 2% O_2_, 5% CO_2_, 93% N_2_; MAP2: 3% O_2_, 7% CO_2_, 90% N_2_.

**Table 9:** Evolution of antioxidant activities in fresh peeled bamboo sprouts packaged using MAP and vacuum systems.

DPPH Test (IC_50_, µg/mL)				
Storage Time (Days)	MAP 1	MAP 2	VACUUM	Sign.
0	24.77 ± 3.14 ^aA^	24.77 ± 3.14 ^aA^	24.77 ± 3.14 ^aA^	ns
7	39.92 ± 0.48 ^eC^	39.02 ± 2.03 ^cB^	44.41 ± 2.03 ^bA^	**
14	37.44 ± 0.27 ^dC^	38.00 ± 1.56 ^cB^	47.10 ± 1.56 ^cA^	**
21	33.26 ± 1.04 ^cC^	34.98 ± 3.37 ^bB^	69.63 ± 3.37 ^dA^	**
28	31.97 ± 0.27 ^bC^	32.74 ± 3.67 ^bB^	71.12 ± 3.67 ^eA^	**
Sign.	**	**	**	
ABTS test (IC_50_, µg/mL)				
Storage time (days)	MAP 1	MAP 2	VACUUM	Sign.
0	0.78 ± 0.04 ^aA^	0.78 ± 0.04 ^aA^	0.78 ± 0.04 ^aA^	ns
7	0.82 ± 0.48 ^bA^	0.89 ± 0.05 ^bA^	0.80 ± 0.53 ^aA^	*
14	0.96 ± 0.27 ^bC^	1.09 ± 0.56 ^cB^	1.84 ± 0.56 ^bA^	**
21	1.29 ± 0.44 ^cB^	1.34 ± 0.37 ^dB^	2.10 ± 3.37 ^A^	**
28	2.27 ± 0.47 ^dC^	3.56 ± 0.67 ^deB^	4.41 ± 3.67 ^cdA^	**
Sign.	**	**	**	
β-Carotene bleaching test (30 min incubation) (IC_50_, µg/mL)				
Storage time (days)	MAP 1	MAP 2	VACUUM	Sign.
0	7.72 ± 1.85 ^aA^	7.72 ± 1.85 ^aA^	7.72 ± 1.85 ^aA^	ns
7	6.30 ± 0.71 ^aC^	8.83 ± 0.97 ^aB^	9.34 ± 0.97 ^aA^	**
14	6.35 ± 0.48 ^bC^	9.27 ± 2.03 ^cB^	12.32 ± 2.02 ^cA^	**
21	9.31 ± 0.37 ^bC^	11.28 ± 1.56 ^cB^	14.35 ± 1.53 ^cA^	**
28	11.35 ± 0.89 ^cC^	13.27 ± 3.27 ^bB^	17.36 ± 3.34 ^bA^	**
Sign.	**	**	**	
β-Carotene bleaching test (60 min incubation) (IC_50_, µg/mL)				
Storage time (days)	MAP 1	MAP 2	VACUUM	Sign.
0	10.54 ± 1.97 ^aA^	10.54 ± 1.97 ^aA^	10.54 ± 1.97 ^aA^	ns
7	7.32 ± 0.71 ^aC^	9.34 ± 0.97 ^aB^	11.35 ± 0.97 ^aA^	**
14	9.27 ± 0.48 ^bC^	11.31 ± 2.03 ^cB^	13.32 ± 2.02 ^cA^	**
21	11.24 ± 0.37 ^bC^	14.28 ± 1.56 ^cB^	16.20 ± 1.53 ^cA^	**
28	14.29 ± 1.00 ^cC^	16.89 ± 3.27 ^bB^	19.31 ± 3.34 ^bA^	**
Sign.	**	**	**	
FRAP test (FRAP value, μM Fe (II)/g)				
Storage time (days)	MAP 1	MAP 2	VACUUM	Sign.
0	14.26 ± 2.75 ^aA^	14.26 ± 2.75 ^aA^	14.26 ± 2.75 ^aA^	ns
7	0.52 ± 0.48 ^bA^	0.30 ± 0.03 ^bB^	0.08 ± 0.03 ^cC^	**
14	0.49 ± 0.27 ^cA^	0.26 ± 0.06 ^bA^	0.09 ± 0.06 ^cB^	*
21	0.47 ± 0.04 ^cA^	0.18 ± 0.37 ^cB^	0.10 ± 0.37 ^bC^	**
28	0.44 ± 0.27 ^cA^	0.14 ± 0.67 ^cB^	0.11 ± 0.67 ^bB^	**
Sign.	**	**	**	

MAP: modified atmosphere packaging. MAP1: 2% O_2_, 5% CO_2_, 93% N_2_; MAP2: 3% O_2_, 7% CO_2_, 90% N_2_. Data are reported as mean ± standard deviation (n = 3). Results followed by different capital letters in the same row indicate differences among the samples. The different lowercase letters in the same column indicate differences during storage. Differences within and between groups were evaluated by a one-way ANOVA followed by Tukey’s test (**, *p* < 0.01). Results followed by different letters are significantly different with * indicating a *p* ≤ 0.05, ** indicating a *p* ≤ 0.01, and ns indicating a *p* > 0.05 which is not significant.

## Data Availability

Data is contained within the article and Supplementary Materials.

## Supplementary Materials

The following supporting information can be downloaded at: https://www.mdpi.com/article/10.3390/antiox13020185/s1, Table S1: Evolution of a_w_ and pH in fresh peeled bamboo sprouts packaged in MAP and VACUUM packaging systems; Table S2: CIELab parameters in bamboo sprouts (apical and basal sections) packaged in MAP and VACUUM packaging systems; Table S3: Evolution of the △E parameter during bamboo sprout storage packaged in MAP and VACUUM packaging systems; Table S4: Total variance explained; Figure S1: Chromatographic profile of fresh bamboo sprouts: 1. Gallic Acid; 2. Protocatheic Acid; 3. Chlorogenic Acid; 4. Caffeic Acid; 5. Ferulic Acid; 6. Isoorientin; 7. Orientin; 8. Isovitexin, 9. *p*-Coumaric Acid; 10 Rutin.
