# Supplementary material for: Evaluation of the Shelf life of Ready-to-Eat Fresh Bamboo Sprouts (Phyllostachys edulis) Packaged in a Modified Atmosphere or Vacuum: A Comparative Study
Source: Antioxidants (Basel). 2024 Feb 1;13(2):185. doi: 10.3390/antiox13020185 (PMC10885971; doi:10.3390/antiox13020185)
Supplement: Supplementary file 1 [file antioxidants-13-00185-s001.zip › antioxidants-2814007-supplementary.pdf]

Supplementary materials

Table S1. Evolution of a_w and pH in fresh peeled bamboo sprouts packaged in MAP and VACUUM packaging systems.

Storage time (days)	MAP 1	MAP 2	VACUUM	Sign.
	a_w	a_w	a_w	
0	0.996 ± 0.002^{aA}	0.996 ± 0.002^{aA}	0.994 ± 0.002^{aA}	ns
7	0.978 ± 0.001^{bcA}	0.968 ± 0.001^{bcAB}	0.966 ± 0.001^{bcB}	**
14	0.971 ± 0.001^{aA}	0.961 ± 0.001^{bA}	0.958 ± 0.001^{cB}	**
21	0.966 ± 0.001^{bAB}	0.956 ± 0.001^{bB}	0.952 ± 0.001^{bC}	**
28	0.957 ± 0.00^{cB}	0.950 ± 0.00^{cC}	0.948 ± 0.00^{cA}	**
Sign.	**	**	**	
	pH	pH	pH	
0	6.36 ± 0.06^{bA}	6.36 ± 0.06^{bA}	6.12 ± 0.06^{bB}	*
7	5.56 ± 0.06^{bA}	5.46 ± 0.06^{bB}	5.26 ± 0.06^{bC}	**
14	5.63 ± 0.06^{abA}	5.53 ± 0.06^{abB}	5.13 ± 0.06^{abC}	**
21	5.68 ± 0.04^{aA}	5.38 ± 0.04^{aB}	5.08 ± 0.04^{aC}	**
28	5.70 ± 0.02^{aA}	5.40 ± 0.02^{aC}	5.22 ± 0.02^{aC}	**
Sign.	**	**	**	

MAP: modified atmosphere packaging. MAP1: 2%O_2, 5%CO_2, 93%N_2; MAP2: 3% O_2, 7% CO_2, 90 % N_2. Data are reported as mean ± standard deviation (n = 3). Differences were evaluated by one-way analysis of variance (ANOVA) test completed with a multicomparison Tukey's test. Means in the same column with different capital letters differ significantly (Sign.) (**p < 0.01), means in the same row with different uppercase differ significantly (**p < 0.01, *p < 0.05); ns: not significant.

Table S2. CIELab parameters in bamboo sprouts (upper and basal sections) packaged in MAP and VACUUM packaging systems.

		Apical section		
a*	MAP1	MAP2	VACUUM	Sign.
T0	3.63±0.71^{aA}	3.63±0.71^{aA}	0.59±0.71^{aB}	**
T7	0.16±0.48^{bB}	0.14±0.48^{bA}	0.57±0.48^{bB}	**
T14	0.11±0.37^{b}	0.10±0.37^{b}	0.58±0.37^{b}	**
T21	0.10±1.00^{bB}	-1.08±1.00^{cB}	0.60±1.00^{cA}	**
T28	0.29±0.22^{bB}	0.25±0.20^{bB}	0.58±0.20^{bA}	**
Sign.	**	**	**	
b*	MAP1	MAP2	VACUUM	Sign.
T0	19.07±0.97^{aA}	19.07±0.97^{aA}	1.97±0.97^{aB}	**
T7	5.82±2.03^{cA}	5.45±2.03^{cB}	1.95±2.03^{cC}	**
T14	5.20±1.56^{cA}	5.00±1.56^{cA}	1.95±1.56^{cB}	**
T21	8.76±3.37^{bA}	8.50±3.27^{bB}	1.97±3.27^{bC}	**
T28	7.21±3.67^{bcA}	7.00±3.67^{bcA}	1.96±3.67^{bcB}	**
Sign.	**	**	**	
		Basal section		
a*	MAP1	MAP2	VACUUM	Sign.
0	3.88±0.60^{aA}	3.88±0.60^{aA}	0.58±0.60^{aB}	**
7	0.12±0.14^{bB}	0.11±0.14^{bB}	0.59±0.14^{bA}	**
14	-1.00±0.40^{cC}	-1.00±0.40^{cA}	0.58±0.40^{cB}	**
21	0.02±0.92^{bB}	0.02±0.92^{bB}	0.61±0.92^{bA}	**
28	-0.03±1.09^{bB}	-0.03±1.09^{bB}	0.57±1.09^{bA}	**
Sign.	**	**	**	
b*	MAP1	MAP2	VACUUM	Sign.
0	19.59±1.06^{aA}	19.59±1.06^{aA}	1.96±1.06^{aB}	**
7	4.30±0.82^{cA}	4.30±0.82^{cB}	1.95±0.82^{cB}	**
14	15.53±3.00^{aA}	14.53±3.00^{aA}	1.94±3.00^{aB}	**
21	9.30±5.40^{bA}	9.12±5.40^{bB}	1.96±5.40^{bC}	**
28	6.78±3.47^{bcA}	6.66±3.47^{bcA}	1.94±3.47^{bcB}	**
Sign.	**	**	**	

MAP: modified atmosphere packaging. MAP1: 2%O_2, 5%CO_2, 93%N_2; MAP2: 3% O_2, 7% CO_2, 90 % N_2. Data are reported as mean ± standard deviation (n = 3). Differences were evaluated by one-way analysis of variance (ANOVA) test completed with a multicomparison Tukey's test. Means in the same column with different capital letters differ significantly (Sign.) (**p < 0.01), means in the same row with different uppercase differ significantly (**p < 0.01).

Table S3. Evolution of △E*ab parameter during bamboo sprouts storage packaged in MAP and VACUUM packaging systems.

△E*ab	MAP1	MAP2	VACUUM	Sign.
0	0.76±0.02^{eB}	0.76±0.03^{eB}	9.9±0.87^{bA}	**
7	10.9±1.88^{bC}	13.88±2,08^{aA}	11.05±1.56^{aB}	**
14	11.06±2.02^{aA}	10±1.76^{bB}	6.48±0.87^{dC}	**
21	2.65±0.06^{dC}	3.31±0.08^{dB}	6.93±0.98^{cA}	**
28	5.62±0.77^{cC}	6.61±0.65^{cB}	6.93±0.34^{cA}	**
Sign.	**	**	**	

MAP: modified atmosphere packaging. MAP1: 2%O_2, 5%CO_2, 93%N_2; MAP2: 3% O_2, 7% CO_2, 90 % N_2. Data are reported as mean ± standard deviation (n = 3). Differences were evaluated by one-way analysis of variance (ANOVA) test completed with a multicomparison Tukey's test. Means in the same column with different capital letters differ significantly (Sign.) (**$p < 0.01$), means in the same row with different uppercase differ significantly (**$p < 0.01$).

Table S4. Total Variance Explained

Component	Initial Eigenvalues			Rotation Sums of Squared Loadings		
	Total	% of Variance	Cumulative %	Total	% of Variance	Cumulative %
1	52.170	74.529	74.529	47.264	67.521	67.521
2	17.830	25.471	100.000	22.736	32.479	100.000

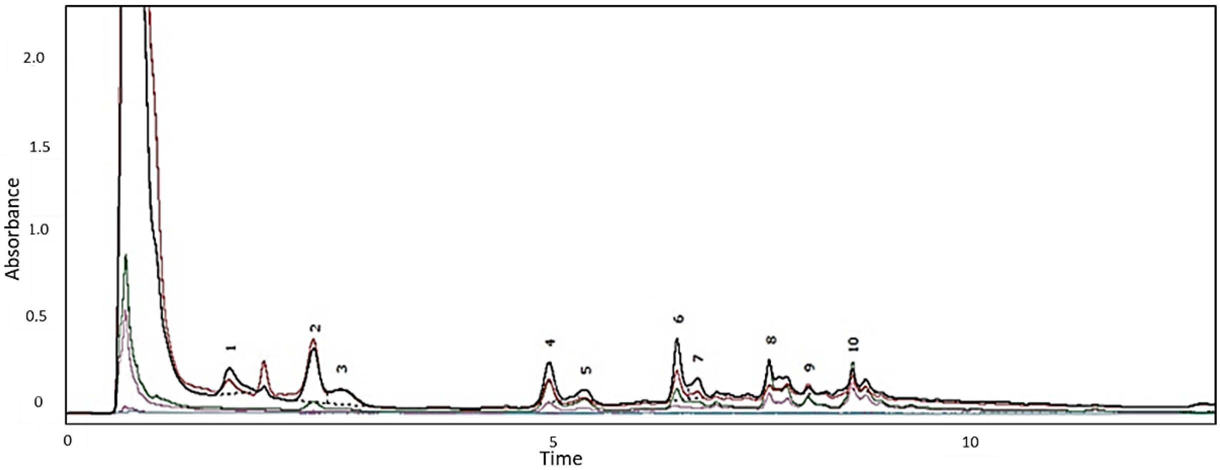

Figure S1. Chromatographic profile of fresh bamboo sprouts: 1 gallic acid; 2 protocatheic; 3 chlorogenic acid; 4 caffeic 5 ferulic 6 isorientin; 7 orientin; 8 isovitexin, 9 p acid; 10 rutin coumaric acid
